# Association of the 2016 US Centers for Disease Control and Prevention Opioid Prescribing Guideline With Changes in Opioid Dispensing After Surgery

**DOI:** 10.1001/jamanetworkopen.2021.11826

**Published:** 2021-06-11

**Authors:** Tori N. Sutherland, Hannah Wunsch, Ruxandra Pinto, Craig Newcomb, Colleen Brensinger, Lakisha Gaskins, Brian T. Bateman, Mark D. Neuman

**Affiliations:** 1 Department of Anesthesiology and Critical Care, Perelman School of Medicine, University of Pennsylvania, Philadelphia; 2 Leonard Davis Institute of Health Economics, University of Pennsylvania, Philadelphia; 3 Center for Perioperative Outcomes Research and Transformation, Perelman School of Medicine, University of Pennsylvania, Philadelphia; 4 Department of Critical Care Medicine, Sunnybrook Health Sciences Centre, Toronto, Ontario, Canada; 5 Department of Anesthesia and Interdepartmental Division of Critical Care Medicine, University of Toronto, Toronto, Ontario, Canada; 6 Center for Clinical Epidemiology and Biostatistics, Perelman School of Medicine, University of Pennsylvania, Philadelphia; 7 Department of Anesthesia, Perioperative, and Pain Medicine, Brigham and Women’s Hospital and Harvard Medical School, Boston, Massachusetts; 8 Division of Pharmacoepidemiology and Pharmacoeconomics, Department of Medicine, Brigham and Women’s Hospital and Harvard Medical School, Boston, Massachusetts; 9 Center for Pharmacoepidemiology Research and Training, University of Pennsylvania Perelman School of Medicine, Philadelphia

## Abstract

**Importance:**

While the 2016 US Centers for Disease Control and Prevention (CDC) guideline for prescribing opioids for chronic pain was not intended to address postoperative pain management, observers have noted the potential for the guideline to have affected postoperative opioid prescribing.

**Objective:**

To assess changes in postoperative opioid dispensing after vs before the CDC guideline release in March 2016.

**Design, Setting, and Participants:**

This cross-sectional study included 361 556 opioid-naive patients who received 1 of 8 common surgical procedures between March 16, 2014, and March 15, 2018. Data were retrieved from a private insurance database, and a retrospective interrupted time series analysis was conducted. Data analysis was conducted from March 2014 to April 2018.

**Exposure:**

Outcomes were measured before and after release of the 2016 CDC guideline.

**Main Outcomes and Measures:**

The primary outcome was the total amount of opioid dispensed in the first prescription filled within 7 days following surgery in morphine milligram equivalents (MMEs); secondary outcomes included the total amount of opioids prescribed and the incidence of any opioid refilled within 30 days after surgery. To characterize absolute opioid dispensing levels, the amount dispensed in initial prescriptions was compared with available procedure-specific recommendations.

**Results:**

The sample included 361 556 opioid-naive patients undergoing 8 general and orthopedic surgical procedures; 164 009 (45.4%) were male patients, and the median (interquartile range) age of the sample was 58 (45 to 69) years. The total amount of opioids dispensed in the first prescription after surgery decreased in the 2 years following the CDC guideline release, compared with an increasing trend in the 2 years prior (prerelease trend: 1.43 MME/month; 95% CI, 0.62 to 2.24 MME/month; *P* = .001; postrelease trend: −2.18 MME/month; 95% CI, −3.01 to −1.35 MME/month; *P* < .001; trend change: −3.61 MME/month; 95% CI, −4.87 to −2.35 MME/month; *P* < .001). Changes in initial dispensing amount trends were greatest for patients undergoing hip or knee replacement (−8.64 MME/month; 95% CI, −11.68 to −5.60 MME/month; *P* < .001). Minimal changes were observed in rates of refills over time (net change: 0.14% per month; 95% CI, 0.06% to 0.23% per month; *P* = .001). Absolute amounts prescribed remained high throughout the period, with nearly half of patients (47.7%; 95% CI, 47.4%-47.9%) treated in the postguideline period receiving at least twice the initial opioid dose anticipated to treat postoperative pain based on available procedure-specific recommendations.

**Conclusions and Relevance:**

In this study, opioid dispensing after surgery decreased substantially after the 2016 CDC guideline release, compared with an increasing trend during the 2 years prior. Absolute amounts prescribed for surgery remained high during the study period, supporting the need for further efforts to improve postoperative pain management.

## Key Points

**Question:**

Was the release of the 2016 US Centers for Disease Control and Prevention (CDC) guideline for prescribing opioids for chronic pain associated with changes in opioid dispensing after surgery?

**Findings:**

In this cross-sectional study of 361 556 opioid-naive patients undergoing 8 common surgical procedures, time-series analysis found that the amount of opioid dispensed after surgery decreased progressively in the 2 years after the guideline release, whereas it was increasing in the 2 years prior.

**Meaning:**

These findings suggest that the release of the 2016 CDC guideline coincided with a decrease in postoperative opioid dispensing across a range of surgical procedures and may have been associated with decreases in overprescribing of opioids for postoperative pain management.

## Teaser

This cross-sectional study assesses changes in postoperative opioid dispensing after vs before the US Centers for Disease Control and Prevention (CDC) guideline for prescribing opioids was released in March 2016.

## Introduction

Opioids are overprescribed after surgery,^1,2^ potentially leading to diversion^3,4^ and other adverse outcomes.^5,6^ In March 2016, the US Centers for Disease Control and Prevention (CDC) released a guideline for opioid prescribing for chronic pain.^7^ While this guideline primarily focused on chronic pain treatment, they also encouraged clinicians to use the lowest effective dosage when prescribing opioids for acute pain treatment and not to prescribe opioids in quantities greater than needed for the expected severity and duration of pain.^7^

The CDC guideline text states that that the acute pain treatment recommendation was not intended to apply to postsurgical pain. However, multiple commentators have reported application beyond the scope of the original guideline, including to acute pain after surgery.^8,9^ The release of the guideline was associated with an acceleration of declining trends in per capita outpatient all-indication opioid dispensing among US patients^10^; however, potential unintended consequences on opioid prescribing for postoperative pain have not been quantified.

To assess changes in postoperative pain treatment associated with the 2016 CDC guideline release, we compared trends in postoperative opioid dispensing among a national cohort of privately insured patients after vs before guideline release. Additionally, we compared the amount dispensed in initial prescriptions with available prescribing recommendations based on the anticipated extent of procedural pain. We hypothesized that the 2016 CDC guideline release would be associated with decreasing postoperative opioid dispensing.

## Methods

### Study Design

In this cross-sectional study, we performed an unadjusted interrupted time series analysis using segmented regression to assess the temporal association of the 2016 CDC opioid prescribing guideline with postoperative opioid dispensing 2 years before and after publication.^11,12^ Interrupted time series analysis is commonly used to assess changes in a continuously measured outcome after the introduction of a new policy or program.^12^ This approach is advantageous for longitudinal data analysis to determine whether a population-level intervention affects outcomes, accounting for secular trends. Here, we assessed whether the CDC guideline release date in March 2016 was associated with changes in postoperative opioid prescribing immediately at the time of release or during the subsequent 2 years. We followed the Strengthening the Reporting of Observational Studies in Epidemiology (STROBE) reporting guideline checklist for observational studies.^13^ This study was determined to be exempt from review by the University of Pennsylvania institutional review board. Because the study used aggregate, deidentified data, informed consent was not required.

### Data Source and Study Sample

We used data from Optum’s deidentified Clinformatics Data Mart Database, a US health insurance database with more than 15 million annual enrollees. It is geographically diverse, including all 50 states, and contains both commercial and Medicare enrollee medical and pharmacy claims data. The study sample included all patients 18 years and older with a claim for any of 8 common general and orthopedic surgical procedures^14^ in the 2 years before or after the guideline publication date (March 16, 2014, through March 15, 2018): breast excision, carpal tunnel release, inguinal hernia repair, knee arthroscopy, laparoscopic appendectomy, laparoscopic cholecystectomy, total hip replacement, and total knee replacement. Procedures were identified using *Current Procedure Terminology* (*CPT*) codes in physician claims (eTable 1 in the Supplement); timing relative to the guideline release date was based on the date of the procedure or hospital discharge, whichever came later. For patients with more than 1 eligible surgery, we used the first procedure. Patients who did not fill an opioid prescription within the first 7 days after surgery, those with claims for more than 1 eligible procedure on the same day, and those who did not have 90 days of continuous enrollment prior to the procedure or admission date (whichever came first) and 30 days after were excluded.

We restricted our sample to patients with no filled opioid prescriptions 90 days prior to surgery (ie, opioid-naive individuals) to capture new opioid prescriptions rather than refills for established chronic pain treatment. We defined baseline comorbidities using *International Classification of Diseases, Ninth Revision (ICD-9)*and *ICD-*10 inpatient and outpatient diagnosis codes in the 90 days prior to surgery using standard crosswalks based on Elixhauser and colleagues’ algorithms.^15,16^ We created indicator variables that corresponded to our 8 surgical procedures and documented inpatient or ambulatory status.

### Outcome Measures

Our primary outcome was the total amount of opioid dispensed in the first prescription filled within 7 days of surgery or hospital discharge, measured in morphine milligram equivalents (MMEs), which we calculated using standard tables.^17^ Secondary outcomes evaluated were total opioids dispensed across all filled prescriptions in MME within 30 days after surgery or discharge, the percentage of patients receiving an opioid refill within 30 days after surgery, the mean MME per day, and the number of days’ supply per month dispensed in the first filled prescription.

We also compared the amount of opioid prescribed with procedure-specific recommendations. We first abstracted prescribing recommendations published by Michigan Opioid Prescribing Engagement Network (OPEN), a resource incorporating literature review, expert opinion, and patient-reported data on postoperative pain and analgesic use that included recommended prescribing ranges for 6 of 8 study procedures; we also compiled 4 available guidelines that included knee arthroscopy and carpal tunnel release (eTable 2 and eTable 3 in the Supplement).^18^ We then calculated the percentages of patients for whom the amount of opioid dispensed in the first filled prescription was greater than and twice the upper bound of anticipated opioid requirements.

### Statistical Analysis

We compared the distributions of procedures and patient characteristics in the periods before vs after guideline release using descriptive statistics and standardized difference (ie, the difference in means or proportions divided by the pooled standard deviation). We considered a standardized difference of less than 0.1 to indicate a comparable distribution between preguideline and postguideline characteristics.^19,20^ We graphed trends in outcomes over time by procedure to visually examine changes in dispensing outcomes.

We performed an unadjusted interrupted time series analysis^12,21^ with segmented regression models for each outcome using monthly repeated measures to quantify differences in postoperative opioid dispensing during 24 months after vs 24 months before guideline release. Analyses examining dispensing relative to Michigan OPEN guidance included only those patients undergoing any of the 6 included procedures; all other analyses contained the full sample. Each regression estimated an aggregated outcome variable measured each month based on 3 independent variables, as follows: (1) the time since the intervention date (in months) to account for secular trends; (2) an indicator of whether the observation occurred before vs after the guideline release date to test for the immediate change at the time of guideline release; and (3) the interaction of month and period (prerelease vs postrelease) to provide an estimate for the difference in preguideline vs postguideline trends (ie, the slope). We visually assessed linearity in trends prior to the date of guideline release. To evaluate for potential first-order autocorrelation of outcome data across months, we used the Durbin-Watson statistic and the proc autoreg function in SAS. We confirmed absence of residual autocorrelation via examination of plotted residuals and the partial autocorrelation function. Analyses were done in SAS version 9.4 (SAS Institute). All tests were 2-sided, and significance was considered at the 5% level.

#### Analyses by Procedure Category

Because we assumed a priori that changes in opioid dispensing over time could differ across procedures associated with more vs less severe postoperative pain, we carried out a multiple-group interrupted time series analysis that added an indicator for total hip or knee arthroplasty vs all other procedures that interacted with all other terms. This grouping was based on visual inspection of initial trends in prescribing outcomes and published guidance recommending higher initial prescribing levels for these procedures.^2,18^

#### Supplemental Analyses

To assess whether opioid dispensing relative to recommendations may have been informed by the specific reference chosen, we compiled prescribing recommendations for all 8 study procedures from 4 separate published guidelines (including Michigan OPEN) and selected the highest referenced dose as the upper recommended limit.^18,22,23,24^ We then quantified the percentage of patients over time who received more than twice the recommended prescribing amount.

We carried out a number of additional regression analyses to assess the robustness of our main findings with different analytic assumptions. These included additional interrupted time series analyses for the primary outcome: (1) stratified by the 8 separate procedures considered here; (2) excluding patients with MME values for the first dispensed prescription greater than the 99th percentile for the full sample; (3) including adjustment for calendar quarter to account for seasonality; (4) allowing for 3-month and 6-month delays between guideline release and implementation by omitting patients whose surgery date fell within the first 3 months or 6 months after guideline release, and (5) stratified by whether the procedure was addressed in the Michigan OPEN guideline. Additionally, as a draft version of the CDC guideline was released in December 2015, we repeated our primary analysis using this month as an alternate release date. We also carried out a patient-level analysis that used multivariable linear regression to test for changes in the primary outcome after vs before guideline release, adjusted for age, sex, Medicare status, procedure type, and comorbidities. Missing data were handled via complete case analysis, and individuals missing covariate data were excluded.

## Results

Our sample included 361 556 opioid-naive patients undergoing 8 general and orthopedic surgical procedures between 2014 and 2018; 164 009 (45.4%) were male patients, and the median (interquartile range) age was 58 (45-69) years. Overall, 165 401 patients (45.7%) underwent surgery prior to the guideline release date, and 196 155 (54.3%) underwent surgery after. We observed modest differences in the percentage of patients with obesity (body mass index, calculated as weight in kilograms divided by height in meters squared, >30) treated in the prerelease vs postrelease periods (24 843 [15.0%] vs 38 370 [19.6%]; standardized difference, 0.12) and the percentage covered by Medicare (48 116 [29.1%] vs 69 057 [35.2%]; standardized difference, 0.13); other patient and procedure characteristics were broadly similar (Table 1). The most commonly performed procedures in each period were laparoscopic cholecystectomy (76 206 [21.1%]), knee arthroscopy (70 891 [19.6%]), and total knee replacement (57 499 [15.9%]).

**Table 1.** Patient and Procedure Characteristics Across Study Periods

Characteristic	Patients by period of surgery, No. (%)		Standardized difference
	Preguideline (n = 165 401)	Postguideline (n = 196 155)	
Age, median (IQR), y	57.0 (44.0-68.0)	59.0 (46.0-69.0)	0.10
Male	76 581 (46.3)	87 428 (44.6)	0.04
Female	88 820 (53.7)	108 727 (55.4)	
Procedure category			
Carpal tunnel release	16 574 (10.0)	20 209 (10.3)	0.009
Breast excision	13 175 (8.0)	15 881 (8.1)	0.005
Laparoscopic appendectomy	13 821 (8.4)	16 207 (8.3)	−0.003
Laparoscopic cholecystectomy	34 573 (20.9)	41 633 (21.2)	0.008
Inguinal hernia repair	16 953 (10.2)	17 627 (9.0)	−0.04
Knee arthroscopy	34 797 (21.0)	36 094 (18.4)	−0.07
Total hip replacement	11 123 (6.7)	15 390 (7.8)	0.04
Total knee replacement	24 385 (14.7)	33 114 (16.9)	0.06
Outpatient surgery	42 367 (25.6)	55 442 (28.3)	0.06
Medicare recipient	48 116 (29.1)	69 057 (35.2)	0.13
Comorbidities			
Depression	15 574 (9.4)	21 046 (10.7)	0.04
Alcohol use disorder	1799 (1.1)	2414 (1.2)	0.01
Drug use disorder	1063 (0.6)	1665 (0.8)	0.02
History of psychoses	541 (0.3)	499 (0.3)	−0.01
Congestive heart failure	3862 (2.3)	5892 (3.0)	0.04
Cardiac arrhythmia	16 731 (10.1)	22 253 (11.3)	0.04
Peripheral vascular disease	5910 (3.6)	9097 (4.6)	0.05
Hypertension, uncomplicated	68 612 (41.5)	88 874 (45.3)	0.08
Hypertension, complicated	5815 (3.5)	10 182 (5.2)	0.08
Chronic pulmonary disease	18 894 (11.4)	24 291 (12.4)	0.03
Diabetes, uncomplicated	22 441 (13.6)	28 539 (14.6)	0.03
Kidney failure	6436 (3.9)	9898 (5.0)	0.06
Coagulopathy	2563 (1.5)	4072 (2.1)	0.04
Obesity	24 843 (15.0)	38 370 (19.6)	0.12

Abbreviation: IQR, interquartile range.

**Figure 1.**
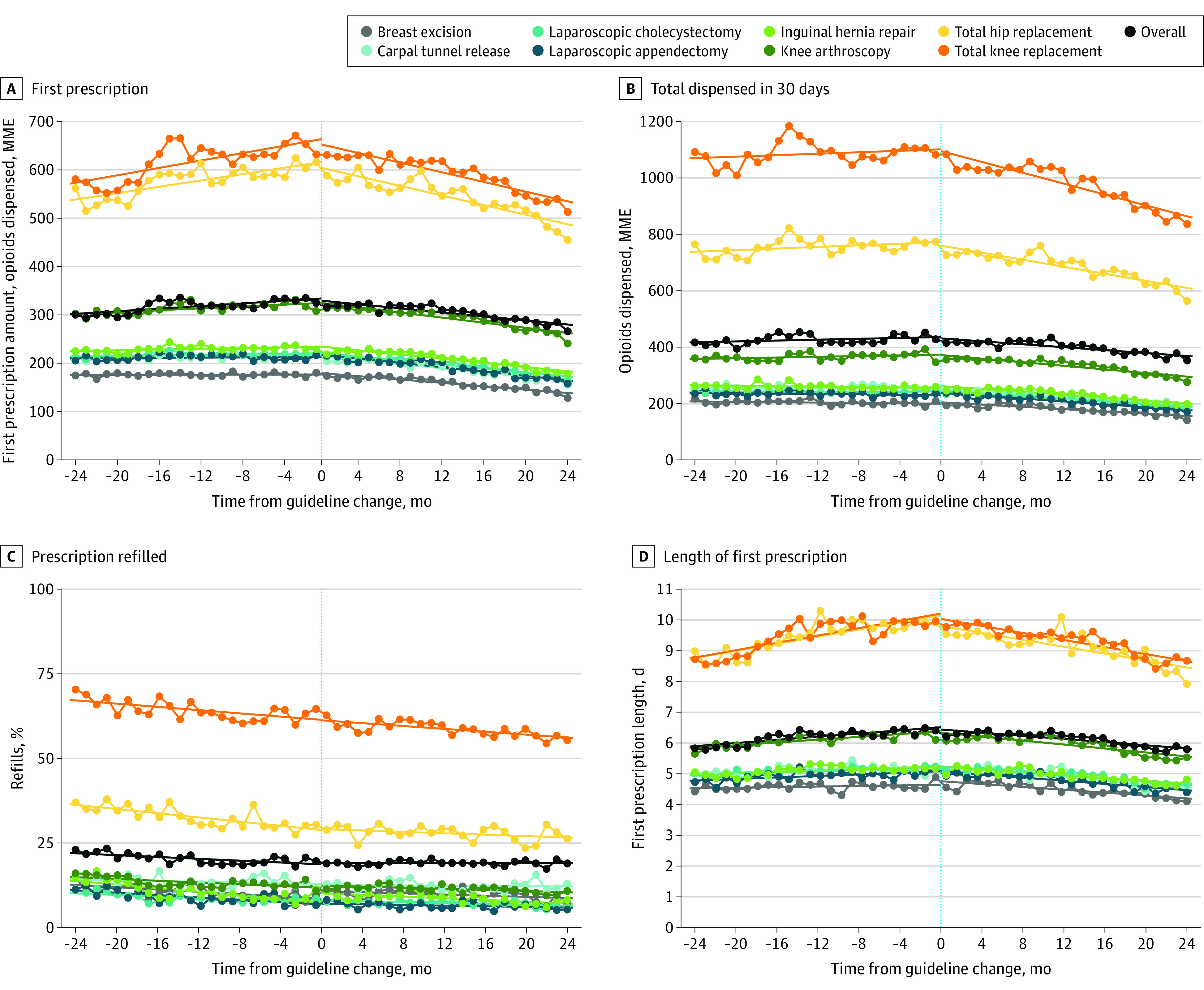
Opioids Prescribed Before and After 2016 US Centers for Disease Control and Prevention Guideline MME indicates morphine milligram equivalent.

During the 24 months prior to guideline release, the mean amount of opioid dispensed in the first filled prescription within 7 days after surgery increased from 301 MME (95% CI, 295-307 MME) to 325 MME (95% CI, 319-331 MME); the mean amount of opioid dispensed in the first 30 days after surgery increased from 416 MME (95% CI, 405-428 MME) to 428 MME (95% CI, 417-438 MME); and the percentage of patients refilling a prescription decreased from 23.0% (95% CI, 22.0%-24.0%) to 19.6% (95% CI, 18.7%-20.6%). During the 24 months following guideline release, the mean amount of opioid received in the first filled prescription decreased from 316 MME (95% CI, 311-322 MME) to 266 MME (95% CI, 260-271 MME); the mean amount of opioids dispensed in the first 30 days after surgery decreased from 415 MME (95% CI, 405-425 MME) to 352 MME (95% CI, 343-361 MME); and the percentage of patients filling a refill prescription was stable at 19.1% (95% CI, 18.2%-19.9%) and 19.0% (95% CI, 18.1%-19.8%). Similar changes in mean days prescribed and mean MME dispensed by day were observed across procedure categories (Figure 1; eFigure 2 in the Supplement).

Our interrupted times series analysis (Table 2) included all patients in the analytic sample. We estimated a cumulative trend change in the first opioid prescription of −3.61 MME/month (95% CI, −4.87 to −2.35 MME/month; *P* < .001) associated with the CDC guideline release, with a similar cumulative change in the mean total 30-day amount prescribed after surgery (−3.52 MME/month; 95% CI, −5.20 to −1.84 MME/month; *P* < .001). The days’ supply of opioids in the initial postoperative prescription decreased by 0.05 days/month (95% CI, −0.07 to −0.04 days/month; *P* < .001). Mean daily MME dispensed in the initial prescription decreased by 0.18 MME/month (95% CI, −0.32 to −0.04 MME/month; *P* = .02) (eTable 4 in the Supplement). While refill rates were comparatively stable, the rate of change after the guideline release date was smaller in magnitude compared with before the guideline release, resulting in a small cumulative increase in refill rate trends of 0.14% per month (95% CI, 0.06% to 0.23% per month; *P* = .001). We did not observe a significant change in the level of the time series (as opposed to the trend) for any of our 3 outcome measures associated with the guideline release.

**Table 2.** Interrupted Time Series Regression Analysis of Perioperative Opioid Prescriptions for 361 556 Opioid-Naive Patients Before and After US Centers for Disease Control Guideline Release on March 16, 2016

Outcome	Preguideline		Postguideline		Change associated with guideline release			
	Slope (95% CI)	*P* value	Slope (95% CI)	*P* value	Immediate change (95% CI)	*P* value	Change in slope (95% CI)	*P* value
First prescription total morphine equivalents, 7 d	1.43 (0.62 to 2.24)	.001	−2.18 (−3.01 to −1.35)	<.001	−3.79 (−17.43 to 9.85)	.59	−3.61 (−4.87 to −2.35)	<.001
Sum of all prescribed morphine equivalents, 30 d	0.86 (−0.27 to 2.00)	.14	−2.66 (−3.80 to −1.52)	<.001	−4.48 (−25.39 to 16.43)	.68	−3.52 (−5.20 to −1.84)	<.001
Patients receiving a refill, 30 d, %	−0.14 (−0.20 to −0.08)	<.001	0.00 (−0.06 to 0.06)	.93	0.28 (−0.86 to 1.42)	.63	0.14 (0.06 to 0.23)	.001

In our multiple-group interrupted time series analysis (Table 3), the monthly change in the amount of opioid dispensed in the initial prescription associated with guideline introduction for patients undergoing hip and knee replacement was −8.64 MME/month (95% CI, −11.68 to −5.60 MME/month; *P* < .001); for patients undergoing 1 of the other 6 study procedures, it was −2.83 MME/month; (95% CI, −3.67 to −2.00 MME/month; *P* < .001). The trend change in average total opioid prescribing during 30 days after hip and knee replacement was −9.70 MME/month; (95% CI, −14.34 to −5.06 MME/month; *P* < .001); for all other procedures, it was −2.76 MME/month (95% CI, −3.59 to −1.92 MME/month; *P* < .001). We observed a small increase in trends for refills dispensed within 30 days for patients undergoing hip or knee arthroplasty (0.14% per month; 95% CI, 0.02% to 0.26% per month; *P* = .03) but not for patients undergoing other procedures (0.09% per month; 95% CI, 0.00% to 0.18% per month; *P* = .06).

**Table 3.** Interrupted Time Series Regression Analysis of Perioperative Opioid Prescriptions for 361 556 Opioid-Naive Patients Before and After US Centers for Disease Control and Prevention Guideline Release on March 16, 2016, by Procedure

Outcome	Preguideline		Postguideline		Change associated with guideline release			
	Slope (95% CI)	*P* value	Slope (95% CI)	*P* value	Immediate change (95% CI)	*P* value	Change in slope (95% CI)	*P* value
First prescription total morphine equivalents, 7 d								
Hip and knee replacement	3.38 (1.52 to 5.24)	<.001	−5.26 (−7.17 to −3.35)	<.001	−4.05 (−30.70 to 22.60)	.77	−8.64 (−11.68 to −5.60)	<.001
All other procedures	0.42 (−0.09 to 0.93)	.11	−2.41 (−2.96 to −1.87)	<.001	0.33 (−7.51 to 8.17)	.94	−2.83 (−3.67 to −2.00)	<.001
Sum of all prescribed morphine equivalents, 30 d								
Hip and knee replacement	0.86 (−2.03 to 3.75)	.56	−8.84 (−11.77 to −5.91)	<.001	−2.50 (−46.46 to 41.46)	.91	−9.70 (−14.34 to −5.06)	<.001
All other procedures	0.00 (−0.54 to 0.53)	.99	−2.76 (−3.32 to −2.21)	<.001	0.92 (−8.19 to 10.03)	.84	−2.76 (−3.59 to −1.92)	<.001
Patients receiving a refill, 30 d, %								
Hip and knee replacement	−0.29 (−0.38 to −0.21)	<.001	−0.15 (−0.24 to −0.07)	.001	−0.18 (−1.87 to 1.50)	.83	0.14 (0.02 to 0.26)	.03
All other procedures	−0.15 (−0.21 to −0.09)	<.001	−0.06 (−0.12 to 0.00)	.05	0.62 (−0.39 to 1.62)	.23	0.09 (0.00 to 0.18)	.06

**Figure 2.**
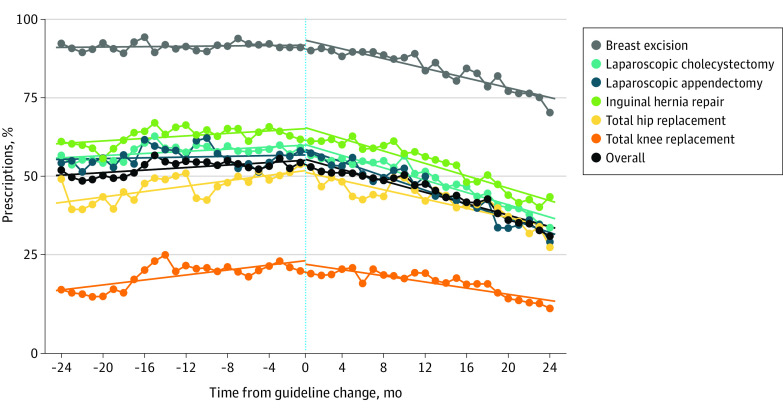
Opioid Prescriptions More Than Twice the Maximum Amount Recommended by Michigan Opioid Prescribing Engagement Network

Finally, we evaluated the fraction of patients for whom the amount of opioid in the initial filled prescription exceeded the amount anticipated to treat postoperative pain based on available procedure-specific guidance. Among the 253 882 patients undergoing any of the 6 procedures addressed by Michigan OPEN^18^ recommendations, the fraction receiving twice the maximum anticipated amount required varied across procedure and decreased progressively overall and within each procedure following the CDC guideline release (Figure 2; eTable 5 in the Supplement). Overall, 89.2% (95% CI, 89.0%-89.3%) of patients undergoing 1 of these 6 procedures after guideline release received an initial opioid prescription amount that was greater than the upper recommended amount, and 47.7% (95% CI, 47.4%-47.9%) received more than twice this amount. We obtained similar results using the full study sample and prescribing recommendations compiled from 4 available guidance documents (eFigure 1 and eTable 6 in the Supplement).^18,22,23,24^ Results were qualitatively similar in the following alternative regression model scenarios: procedure-specific (eTable 7 in the Supplement), outlier exclusion (eTable 8 in the Supplement), seasonality (eTable 9 in the Supplement), 3-month and 6-month diffusion delays (eTable 10 and eTable 11 in the Supplement), stratification by Michigan OPEN procedures (eTable 12 in the Supplement), alternative start date (eTable 13 in the Supplement), and patient-level multivariable regression (eTable 14 in the Supplement).

## Discussion

In this analysis of 361 556 patients undergoing 8 common surgical procedures, we found that the 2016 CDC guideline release was associated with decreases in postoperative opioid dispensing. While we observed a trend toward increasing amounts of opioids dispensed prior to the guideline, trends in the amount of opioid dispensed in the initial prescription and all prescriptions filled within 30 days after surgery decreased after guideline release. We estimate the CDC prescribing guideline was associated with a clinically meaningful decrease of approximately 3.61 MME/month and 0.05 days’ supply in the initial prescription, equivalent to a reduction of approximately 11 oxycodone 5-mg pills during the 2 years after guideline release. Absolute prescribing levels remained high, with nearly half of all patients treated after March 2016 receiving amounts more than 2 times greater than the levels anticipated to be necessary. Our results were consistent across a number of supplemental analyses, including a patient-level regression that adjusted for potential confounders.

While unintended, multiple past observers have noted the potential for the 2016 CDC guideline to influence postoperative pain treatment. A 2017 consensus panel convened by the American Academy of Pain Medicine Foundation observed potential for the guideline to “promote unintended ‘drift’ into an increasing reluctance by providers to prescribe opioids” after surgery.^9^ A subsequent report by the CDC guideline’s authors noted that “practices purportedly derived from the guideline have … been inconsistent with, and often go beyond, its recommendations,”^8^ including application of the guideline’s acute pain treatment recommendations to postsurgical prescribing contexts. Indeed, our findings suggest that the CDC guideline may have contributed to changes in postsurgical pain treatment, even despite its exclusion from the guideline’s stated scope.^7^ Such changes may also have been driven in part by concurrent local or regional initiatives, such as a 2015 Washington State guideline focused on limiting opioid overprescribing after surgery,^25^ as well as by broader secular forces, potentially including an emerging awareness among clinicians and patients in general regarding opioid-related harms. While the specific approach used here does not allow us to disentangle the potential impact of the CDC guideline vs other secular forces, this topic remains important for further study.

Our work extends prior evaluations of the guideline’s associations with changes in practice. A 2018 analysis using similar methods found that the release coincided with an acceleration of a prior trend toward decreasing numbers and quantity of all-indication opioid prescriptions per capita.^10^ Consistent with our prior work,^26^ we found the opioid quantity dispensed after surgery continued to increase before guideline release, followed by a change in prescribing trajectory after release. Additionally, our analysis suggests that, while unintended, changes in surgical prescribing associated with guideline release were likely to have had beneficial effects overall by reducing excess opioid dispensing. The lack of a marked difference in refill rates provides reassurance that the observed dispensing decreases did not meaningfully result in additional patient care needs. Moreover, as dispensed opioid quantity remained high, it is unlikely that guideline misapplication led to significant opioid underprescribing for surgical pain.

### Limitations

This study has limitations. Our data come from a private insurance claims database and may not be generalizable to other contexts; additionally, given that the database did not include data on prescriptions that may have been issued but not filled, our work may not fully capture changes in physician prescribing behavior. Since we did not have access to measures of pain, our analysis is limited in its ability to speak to the potential impact on patient-centered outcomes. Because our analysis does not include a concurrent control group, it is limited in its ability to exclude the potential association between secular prescribing trends and our findings. Time series analysis findings may be affected by confounders that vary over time, although we were able to confirm our findings in a patient-level regression that accounted for a range of potential confounders.

## Conclusions

This study has important policy and practice implications. Although the primary focus of the 2016 CDC guideline was on opioid prescribing for chronic pain, our findings suggest that the guideline was associated with clinically relevant changes in patterns of opioid prescribing after surgery. Concurrently, our observation of high opioid amounts dispensed relative to recommendations indicates ongoing opportunities to improve pain treatment after surgery and the potential need for additional policy and clinical interventions to transform care.

## Notes

Supplement. **eTable 1.** Procedures Included in the Analysis and Associated *CPT* Codes **eTable 2.** Michigan OPEN Recommended Postoperative Opioid Prescribing Ranges for 6 Study Procedures **eTable 3.** Summarized Recommended Postoperative Opioid Prescribing Ranges for 8 Study Procedures, Abstracted from 4 Available Guidelines **eTable 4.** Interrupted Time Series (ITS) Regression Analysis of (1) Days Supplied in Initial Filled Prescription; (2) Initial Filled Prescription MME per Day (N = 361 556) **eTable 5. **Interrupted Time Series Analysis: Percentage of Patients Receiving at Least 2 Times the Maximum Postoperative Opioid Amount Recommended by Michigan OPEN Across 6 Procedures Before and After the 2016 CDC Guideline Release (N = 253 882) **eFigure 1.** Percentage of Patients by Month Undergoing Any of 8 Study Procedures Receiving at Least 2 Times the Maximum Postoperative Opioid Amount Recommended Across 4 Available Resources Before and After the 2016 CDC Guideline Release **eTable 6.** Interrupted Time Series (ITS) Analysis: Percentage of Patients Undergoing Any of 8 Study Procedures Receiving at Least 2 Times the Maximum Postoperative Opioid Amount Recommended by 4 Available Resources Before and After the 2016 CDC Guideline Release (N = 361 556) **eTable 7.** Interrupted Time Series (ITS) Regression Analysis of First Prescription Total Morphine Equivalents Before and After CDC Guideline Release, by Surgery Type (N = 361 556) **eTable 8.** Interrupted Time Series (ITS) Regression Analysis of First Prescription Total Morphine Equivalents Before and After CDC Guideline Release, Excluding Values Greater Than the 99th Percentile (N = 358 775) **eTable 9. **Interrupted Time Series (ITS) Regression Analysis of First Prescription Total Morphine Equivalents Before and After CDC Guideline Release, Adjusted for Calendar Quarter (N = 361 556) **eTable 10.** Interrupted Time Series (ITS) Regression Analysis of First Prescription Total Morphine Equivalents Before and After CDC Guideline Release, 3-Month Diffusion Delay (N = 338 188) **eTable 11.** Interrupted Time Series (ITS) Regression Analysis of First Prescription Total Morphine Equivalents Before and After CDC Guideline Release, 6-Month Diffusion Delay (N = 315 385) **eTable 12.** Interrupted Time Series (ITS) Regression Analysis of First Prescription Total Morphine Equivalents Before and After CDC Guideline Release, Stratified by Procedures Included in the Michigan OPEN Recommendations (N = 253 882) vs Non–Michigan OPEN Procedures (N = 107 674) **eTable 13.** Interrupted Time Series (ITS) Regression Analysis of First Prescription Total Morphine Equivalents Before and After CDC Guideline Release, Alternative Start Date of December 15, 2015 (N = 361 556) **eTable 14.** Patient-Level Multivariable Linear Regression to Estimate First Prescription Total Morphine Equivalents Before and After CDC Guideline Release, Adjusted for Age, Gender, and Comorbidities (N = 361 534) **eFigure 2.** Average MME Dispensed, by Day, in the Initial Prescription Within 7 Days of Surgery eReferences.
