# Supplementary material for: Association of the 2016 US Centers for Disease Control and Prevention Opioid Prescribing Guideline With Changes in Opioid Dispensing After Surgery
Source: JAMA Netw Open. 2021 Jun 11;4(6):e2111826. doi: 10.1001/jamanetworkopen.2021.11826 (PMC8196343; doi:10.1001/jamanetworkopen.2021.11826)
Supplement: Supplement. — eTable 1. Procedures Included in the Analysis and Associated CPT Codes eTable 2. Michigan OPEN Recommended Postoperative Opioid Prescribing Ranges for 6 Study Procedures eTable 3. Summarized Recommended Postoperative Opioid Prescribing Ranges for 8 Study Procedures, Abstracted from 4 Available Guidelines eTable 4. Interrupted Time Series (ITS) Regression Analysis of (1) Days Supplied in Initial Filled Prescription; (2) Initial Filled Prescription MME per Day (N = 361 556) eTable 5. Interrupted Time Series Analysis: Percentage of Patients Receiving at Least 2 Times the Maximum Postoperative Opioid Amount Recommended by Michigan OPEN Across 6 Procedures Before and After the 2016 CDC Guideline Release (N = 253 882) eFigure 1. Percentage of Patients by Month Undergoing Any of 8 Study Procedures Receiving at Least 2 Times the Maximum Postoperative Opioid Amount Recommended Across 4 Available Resources Before and After the 2016 CDC Guideline Release eTable 6. Interrupted Time Series (ITS) Analysis: Percentage of Patients Undergoing Any of 8 Study Procedures Receiving at Least 2 Times the Maximum Postoperative Opioid Amount Recommended by 4 Available Resources Before and After the 2016 CDC Guideline Release (N = 361 556) eTable 7. Interrupted Time Series (ITS) Regression Analysis of First Prescription Total Morphine Equivalents Before and After CDC Guideline Release, by Surgery Type (N = 361 556) eTable 8. Interrupted Time Series (ITS) Regression Analysis of First Prescription Total Morphine Equivalents Before and After CDC Guideline Release, Excluding Values Greater Than the 99th Percentile (N = 358 775) eTable 9. Interrupted Time Series (ITS) Regression Analysis of First Prescription Total Morphine Equivalents Before and After CDC Guideline Release, Adjusted for Calendar Quarter (N = 361 556) eTable 10. Interrupted Time Series (ITS) Regression Analysis of First Prescription Total Morphine Equivalents Before and After CDC Guideline Release, 3-Month Diffusion Delay (N = 33 [file jamanetwopen-e2111826-s001.pdf]

Supplemental Online Content

Sutherland TN, Wunsch H, Pinto R, et al. Association of the 2016 US Centers for Disease Control and Prevention opioid prescribing guideline with changes in opioid dispensing after surgery. *JAMA Netw Open.* 2021;4(6):e2111826. doi:10.1001/jamanetworkopen.2021.11826

eTable 1. Procedures Included in the Analysis and Associated *CPT* Codes
eTable 2. Michigan OPEN Recommended Postoperative Opioid Prescribing Ranges for 6 Study Procedures
eTable 3. Summarized Recommended Postoperative Opioid Prescribing Ranges for 8 Study Procedures, Abstracted from 4 Available Guidelines
eTable 4. Interrupted Time Series (ITS) Regression Analysis of (1) Days Supplied in Initial Filled Prescription; (2) Initial Filled Prescription MME per Day (N = 361 556)
eTable 5. Interrupted Time Series Analysis: Percentage of Patients Receiving at Least 2 Times the Maximum Postoperative Opioid Amount Recommended by Michigan OPEN Across 6 Procedures Before and After the 2016 CDC Guideline Release (N = 253 882)
eFigure 1. Percentage of Patients by Month Undergoing Any of 8 Study Procedures Receiving at Least 2 Times the Maximum Postoperative Opioid Amount Recommended Across 4 Available Resources Before and After the 2016 CDC Guideline Release
eTable 6. Interrupted Time Series (ITS) Analysis: Percentage of Patients Undergoing Any of 8 Study Procedures Receiving at Least 2 Times the Maximum Postoperative Opioid Amount Recommended by 4 Available Resources Before and After the 2016 CDC Guideline Release (N = 361 556)
eTable 7. Interrupted Time Series (ITS) Regression Analysis of First Prescription Total Morphine Equivalents Before and After CDC Guideline Release, by Surgery Type (N = 361 556)
eTable 8. Interrupted Time Series (ITS) Regression Analysis of First Prescription Total Morphine Equivalents Before and After CDC Guideline Release, Excluding Values Greater Than the 99th Percentile (N = 358 775)
eTable 9. Interrupted Time Series (ITS) Regression Analysis of First Prescription Total Morphine Equivalents Before and After CDC Guideline Release, Adjusted for Calendar Quarter (N = 361 556)
eTable 10. Interrupted Time Series (ITS) Regression Analysis of First Prescription Total Morphine Equivalents Before and After CDC Guideline Release, 3-Month Diffusion Delay (N = 338 188)
eTable 11. Interrupted Time Series (ITS) Regression Analysis of First Prescription Total Morphine Equivalents Before and After CDC Guideline Release, 6-Month Diffusion Delay (N = 315 385)
eTable 12. Interrupted Time Series (ITS) Regression Analysis of First Prescription Total Morphine Equivalents Before and After CDC Guideline Release, Stratified by Procedures Included in the Michigan OPEN Recommendations (N = 253 882) vs Non–Michigan OPEN Procedures (N = 107 674)
eTable 13. Interrupted Time Series (ITS) Regression Analysis of First Prescription Total Morphine Equivalents Before and After CDC Guideline Release, Alternative Start Date of December 15, 2015 (N = 361 556)

© 2021 Sutherland TN et al. *JAMA Network Open.*

eTable 14. Patient-Level Multivariable Linear Regression to Estimate First Prescription Total Morphine Equivalents Before and After CDC Guideline Release, Adjusted for Age, Gender, and Comorbidities (N = 361 534)
eFigure 2. Average MME Dispensed, by Day, in the Initial Prescription Within 7 Days of Surgery
eReferences.

This supplemental material has been provided by the authors to give readers additional information about their work.

© 2021 Sutherland TN et al. *JAMA Network Open.*

eTable 1. Procedures Included in the Analysis and Associated *CPT* Codes

**Procedure name**	**CPT Codes**
General surgical procedures	
Breast excision	19301, 19302, 19120
Laparoscopic Cholecystectomy	47562; 47563; 47564
Inguinal Hernia Repair	49505; 49507; 49520; 49521; 49525
Laparoscopic Appendectomy	44970
Orthopedic surgical procedures	
Total Knee Replacement	27446;27447; 27486; 27487
Total Hip Replacement	27130; 27132*
Carpal Tunnel Release	64721; 29848
Knee Arthroscopy	29881; 29880; 29877; 29875; 29876; 29870

*Excluding any patient with an International Classification of Diseases 9th or 10th Revision, Clinical Modification diagnosis code indicating hip fracture.

© 2021 Sutherland TN et al. *JAMA Network Open.*

eTable 2. Michigan OPEN Recommended Postoperative Opioid Prescribing Ranges for 6 Study Procedures.[3] Prescribing ranges for six of eight study procedures are specified in Michigan OPEN guidance (breast excision; laparoscopic Appendectomy; laparoscopic cholecystectomy; inguinal hernia repair; hip arthroplasty; knee arthroplasty); two study procedures (carpal tunnel release and knee arthroscopy) are not specified in the Michigan OPEN guidance.

	Oxycodone 5mg Tabs (N), Range	Maximum Total Dose Oxycodone (Mg)	Morphine Milligram Equivalents (MMEs), Maximum Dose
**Carpal Tunnel Release**	N/A	N/A	N/A
**Breast Excision**	0-5	25	37.5
**Laparoscopic Appendectomy**	0-10	50	75
**Laparoscopic Cholecystectomy**	0-10	50	75
**Inguinal Hernia Repair**	0-10	50	75
**Knee Arthroscopy**	N/A	N/A	N/A
**Hip Arthroplasty**	0-30	150	225
**Knee Arthroplasty**	0-50	250	375

© 2021 Sutherland TN et al. *JAMA Network Open.*

eTable 3. Summarized Recommended Postoperative Opioid Prescribing Ranges for 8 Study Procedures, Abstracted from 4 Available Guidelines

Procedure	Maximum Recommended Opioid Amount at Discharge			
	Maximum Number Oxycodone 5mg tabs	Maximum Total Dose Oxycodone (Mg)	Total Morphine Milligram Equivalents	Source
Carpal Tunnel Release	5-10	50	75	[1]
Breast Excision	5-10	50	75	[2,3,4]
Laparoscopic Appendectomy	10	50	75	[3]
Laparoscopic Cholecystectomy	10-15	75	112.5	[2,3,4]
Inguinal Hernia Repair	10-15	75	112.5	[2,3,4]
Knee Arthroscopy	10	10	75	[2]
Hip Arthroplasty	30	150	225	[3]
Knee Arthroplasty	50	250	375	[3]

**eTable 4.** Interrupted Time Series (ITS) Regression Analysis of (1) Days Supplied in Initial Filled Prescription; (2) Initial Filled Prescription MME per Day (N = 361 556)								
	**Pre-guideline**		**Post-guideline**		**Change Associated With Guideline Release**			
	**Slope (95% CI)**	**P value**	**Slope (95% CI)**	**P value**	**Immediate change (95% CI)**	**P value**	**Change in Slope (95% CI)**	**P value**
**Model 1: Days Supplied in Initial Prescription**	0.03 (0.02, 0.04)	<0.0001	-0.02 (-0.03, -0.01)	<0.0001	-0.10 (-0.28, 0.08)	0.27	-0.05 (-0.07, -0.04)	<0.0001
**Model 2: Initial Prescription MME per Day**	-0.02 (-0.10, 0.06)	0.63	-0.20 (-0.29, -0.11)	0.0001	0.12 (-1.20, 1.43)	0.86	-0.18 (-0.32, -0.04)	0.02

© 2021 Sutherland TN et al. *JAMA Network Open.*

eTable 5. Interrupted Time Series Analysis: Percentage of Patients Receiving at Least 2 Times the Maximum Postoperative Opioid Amount Recommended by Michigan OPEN Across 6 Procedures Before and After the 2016 CDC Guideline Release (N = 253 882)

Sample	Pre-Guideline		Post-Guideline		Change Associated With Guideline Release			
	Slope (95% CI)	P value	Slope (95% CI)	P value	Immediate change (95% CI)	P value	Change in Slope (95% CI)	P value
**Single-Group Interrupted Time Series: All Study Procedures**								
**All Procedures**	0.19 (0.01, 0.37)	0.04	-0.87 (-1.05, -0.68)	<0.001	0.23 (-2.38, 2.84)	0.87	-1.06 (-1.35, -0.76)	<0.001
**Multiple-Group Interrupted Time Series: Hip or Knee Replacement vs. All Other Procedures**								
**Hip or Knee Replacement**	0.36 (0.14, 0.59)	0.003	-0.58 (-0.81, -0.35)	<0.001	-0.14 (-3.30, 3.02)	0.93	-0.95 (-1.31, -0.58)	<0.001
**All Other Procedures**	0.17 (-0.01, 0.35)	0.08	-0.89 (-1.08, -0.70)	<0.001	-0.37 (-2.64, 1.90)	0.75	-1.06 (-1.37, -0.75)	<0.001

© 2021 Sutherland TN et al. *JAMA Network Open.*

eFigure 1. Percentage of Patients by Month Undergoing Any of 8 Study Procedures Receiving at Least 2 Times the Maximum Postoperative Opioid Amount Recommended Across 4 Available Resources Before and After the 2016 CDC Guideline Release

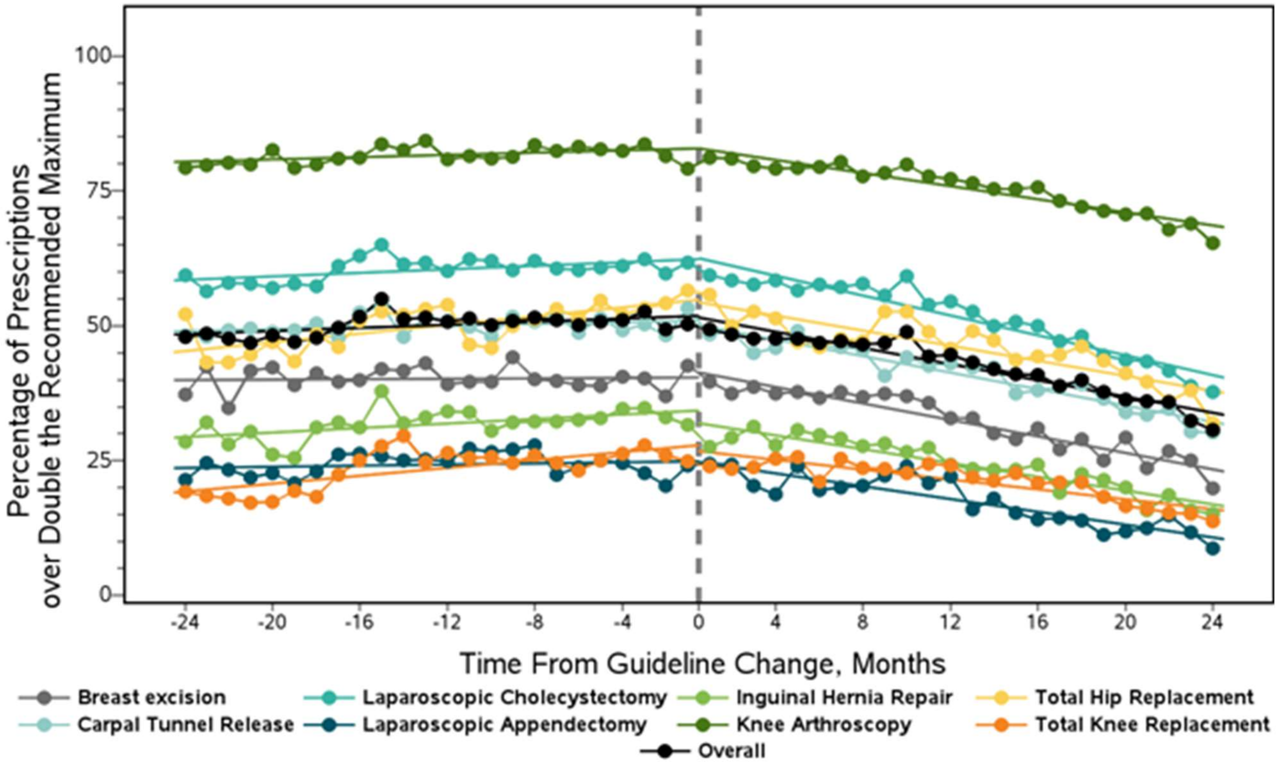

The dashed vertical line corresponds to the date of guideline release (March 16, 2016). The panel shows the percentage of patients overall and in each surgery category who received an amount of opioid in the first opioid prescription filled within 7 days after surgery exceeding two times the highest amount recommended across 4 published resources. [1-4]

© 2021 Sutherland TN et al. *JAMA Network Open.*

eTable 6. Interrupted Time Series (ITS) Analysis: Percentage of Patients Undergoing Any of 8 Study Procedures Receiving at Least 2 Times the Maximum Postoperative Opioid Amount Recommended by 4 Available Resources Before and After the 2016 CDC Guideline Release (N = 361 556)

**Sample**	**Pre-Guideline**		**Post-Guideline**		**Change Associated With Guideline Release**			
	**Slope (95% CI)**	**P value**	**Slope (95% CI)**	**P value**	**Immediate change (95% CI)**	**P value**	**Change in Slope (95% CI)**	**P value**
**Single-Group Interrupted Time Series: All Study Procedures**								
**All Procedures**	0.16 (0.00, 0.31)	0.06	-0.77 (-0.94, -0.61)	<0.001	0.07 (-2.56, 2.69)	0.96	-0.81 (-1.03, -0.58)	<0.001
**Multiple-Group Interrupted Time Series: Hip or Knee Replacement vs. All Other Procedures**								
**Hip or Knee Replacement**	0.36 (0.14, 0.59)	0.003	-0.58 (-0.81, -0.35)	<0.001	-0.14 (-3.30, 3.02)	0.93	-0.95 (-1.31, -0.58)	<0.001
**All Other Procedures**	0.12 (-0.03, 0.27)	0.12	-0.78 (-0.94, -0.62)	<0.001	-0.06 (-2.69, 2.56)	0.96	-0.90 (-1.14, -0.67)	<0.001

© 2021 Sutherland TN et al. *JAMA Network Open.*

eTable 7. Interrupted Time Series (ITS) Regression Analysis of First Prescription Total Morphine Equivalents Before and After CDC Guideline Release, by Surgery Type (N = 361 556)

Surgery	Pre-guideline		Post-guideline		Change Associated With Guideline Release			
	Slope (95% CI)	P value	Slope (95% CI)	P value	Immediate change (95% CI)	P value	Change in Slope (95% CI)	P value
**Breast excision**	0.05 (-0.20, 0.29)	0.71	-1.73 (-1.98, -1.49)	<0.0001	2.89 (-1.94, 7.71)	0.25	-1.78 (-2.13, -1.43)	<0.0001
**Carpal Tunnel**	0.32 (0.01, 0.63)	0.05	-2.11 (-2.42, -1.80)	<0.0001	-1.73 (-7.82, 4.36)	0.58	-2.43 (-2.86, -1.99)	<0.0001
**Laparoscopic cholecystectomy**	0.39 (-0.02, 0.80)	0.07	-2.05 (-2.47, -1.63)	<0.0001	-0.61 (-7.45, 6.22)	0.86	-2.44 (-3.09, -1.79)	<0.0001
**Laparoscopic appendectomy**	0.11 (-0.25, 0.47)	0.54	-2.26 (-2.62, -1.90)	<0.0001	5.56 (-1.45, 12.56)	0.13	-2.38 (-2.89, -1.86)	<0.0001
**Inguinal hernia repair**	0.44 (-0.03, 0.90)	0.08	-2.10 (-2.57, -1.62)	<0.0001	-1.13 (-9.58, 7.31)	0.79	-2.53 (-3.24, -1.83)	<0.0001
**Knee arthroscopy**	0.89 (0.16, 1.61)	0.02	-2.86 (-3.63, -2.09)	<0.0001	4.90 (-8.99, 18.78)	0.49	-3.75 (-4.82, -2.67)	<0.0001
**Hip arthroplasty**	3.34 (1.79, 4.89)	0.0001	-5.03 (-6.59, -3.46)	<0.0001	-11.46 (-39.26, 16.35)	0.42	-8.37 (-10.71, -6.03)	<0.0001
**Knee arthroplasty**	3.51 (1.49, 5.52)	0.001	-5.29 (-7.34, -3.25)	<0.0001	0.24 (-31.83, 32.32)	0.99	-8.80 (-11.98, -5.61)	<0.0001

© 2021 Sutherland TN et al. *JAMA Network Open.*

**eTable 8.** Interrupted Time Series (ITS) Regression Analysis of First Prescription Total Morphine Equivalents Before and After CDC Guideline Release, Excluding Values Greater Than the 99th Percentile (N = 358 775)							
**Pre-guideline**		**Post-guideline**		**Change Associated With Guideline Release**			
**Slope (95% CI)**	**P value**	**Slope (95% CI)**	**P value**	**Immediate change (95% CI)**	**P value**	**Change in Slope (95% CI)**	**P value**
1.36 (0.65, 2.07)	0.001	-1.89 (-2.61, -1.17)	<0.0001	-4.27 (-16.85, 8.31)	0.51	-3.25 (-4.33, -2.18)	<0.0001

© 2021 Sutherland TN et al. *JAMA Network Open.*

eTable 9. Interrupted Time Series (ITS) Regression Analysis of First Prescription Total Morphine Equivalents Before and After CDC Guideline Release, Adjusted for Calendar Quarter (N = 361 556)

**Pre-guideline**		**Post-guideline**		**Change Associated With Guideline Release**			
**Slope (95% CI)**	**P value**	**Slope (95% CI)**	**P value**	**Immediate change (95% CI)**	**P value**	**Change in Slope (95% CI)**	**P value**
1.46 (0.60, 2.33)	0.002	-2.13 (-3.01, -1.25)	<0.0001	-5.13 (-19.05, 8.78)	0.47	-3.60 (-4.96, -2.23)	<0.0001

© 2021 Sutherland TN et al. *JAMA Network Open.*

**eTable 10.** Interrupted Time Series (ITS) Regression Analysis of First Prescription Total Morphine Equivalents Before and After CDC Guideline Release, 3-Month Diffusion Delay (N = 338 188)							
**Pre-guideline**		**Post-guideline**		**Change Associated With Guideline Release**			
**Slope (95% CI)**	**P value**	**Slope (95% CI)**	**P value**	**Immediate change (95% CI)**	**P value**	**Change in Slope (95% CI)**	**P value**
1.35 (0.59, 2.11)	0.001	-2.66 (-3.59, -1.73)	<0.0001	6.22 (-9.28, 21.71)	0.44	-4.01 (-5.30, -2.72)	<0.0001

© 2021 Sutherland TN et al. *JAMA Network Open.*

**eTable 11.** Interrupted Time Series (ITS) Regression Analysis of First Prescription Total Morphine Equivalents Before and After CDC Guideline Release, 6-Month Diffusion Delay (N = 315 385)							
**Pre-guideline**		**Post-guideline**		**Change Associated With Guideline Release**			
**Slope (95% CI)**	**P value**	**Slope (95% CI)**	**P value**	**Immediate change (95% CI)**	**P value**	**Change in Slope (95% CI)**	**P value**
1.34 (0.62, 2.07)	0.0009	-3.23 (-4.30, -2.15)	<0.0001	16.87 (-0.84, 34.57)	0.07	-4.23 (-5.99, -2.47)	<0.0001

© 2021 Sutherland TN et al. *JAMA Network Open.*

**eTable 12.** Interrupted Time Series (ITS) Regression Analysis of First Prescription Total Morphine Equivalents Before and After CDC Guideline Release, Stratified by Procedures Included in the Michigan OPEN Recommendations (N = 253 882) vs Non–Michigan OPEN Procedures (N = 107 674)								
	**Pre-guideline**		**Post-guideline**		**Change Associated With Guideline Release**			
	**Slope (95% CI)**	**P value**	**Slope (95% CI)**	**P value**	**Immediate change (95% CI)**	**P value**	**Change in Slope (95% CI)**	**P value**
**Michigan OPEN Procedures**	1.69 (0.68, 2.69)	0.002	-2.01 (-3.02, -0.99)	0.0004	-5.63 (-23.07, 11.80)	0.53	-3.69 (-5.23, -2.15)	<0.0001
**Non-Michigan OPEN Procedures**	0.65 (0.09, 1.22)	0.03	-2.79 (-3.39, -2.19)	<0.0001	1.39 (-9.46, 12.23)	0.80	-3.44 (-4.29, -2.60)	<0.0001

© 2021 Sutherland TN et al. *JAMA Network Open.*

**eTable 13.** Interrupted Time Series (ITS) Regression Analysis of First Prescription Total Morphine Equivalents Before and After CDC Guideline Release, Alternative Start Date of December 15, 2015 (N = 361 556)							
**Pre-guideline**		**Post-guideline**		**Change Associated With Guideline Release**			
**Slope (95% CI)**	**P value**	**Slope (95% CI)**	**P value**	**Immediate change (95% CI)**	**P value**	**Change in Slope (95% CI)**	**P value**
1.65 (0.68, 2.61)	0.002	-2.08 (-2.78, -1.38)	<0.0001	1.98 (-11.38, 15.35)	0.77	-3.73 (-5.03, -2.42)	<0.0001

© 2021 Sutherland TN et al. *JAMA Network Open.*

eTable 14. Patient-Level Multivariable Linear Regression to Estimate First Prescription Total Morphine Equivalents Before and After CDC Guideline Release, Adjusted for Age, Gender, and Comorbidities (N = 361 534)

Pre-guideline		Post-guideline		Change Associated With Guideline Release			
**Slope (95% CI)**	**P value**	**Slope (95% CI)**	**P value**	**Immediate change (95% CI)**	**P value**	**Change in Slope (95% CI)**	**P value**
1.16 (1.02, 1.29)	<0.0001	-2.83 (-2.96, -2.71)	<0.0001	0.76 (-1.83, 3.36)	0.57	-3.99 (-4.18, -3.80)	<0.0001

1. We included all demographic covariates listed in Table 1, including age in years, gender (22 patients with missing values excluded from analysis), surgery type and all major comorbidities (depression, alcohol abuse, drug abuse, history of psychoses, congestive heart failure, cardiac arrhythmia, peripheral vascular disease, hypertension- complicated and uncomplicated, chronic pulmonary disease, uncomplicated diabetes, renal failure, coagulopathy and obesity).The model controlled for month with relation to the intervention and contained indicators for period (pre- versus post-release), and the interaction of month and period, adjusted for the covariates listed above.

© 2021 Sutherland TN et al. *JAMA Network Open.*

eFigure 2. Average MME Dispensed, by Day, in the Initial Prescription Within 7 Days of Surgery

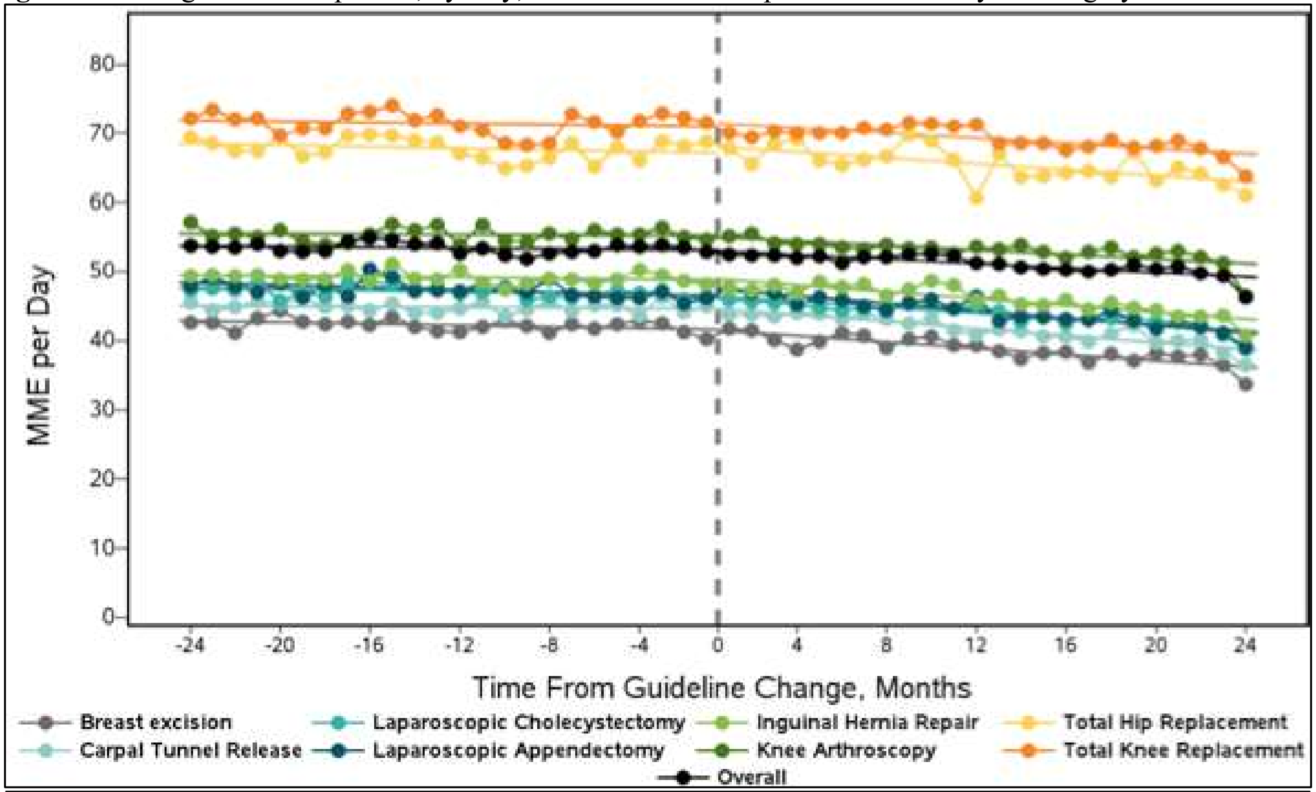

The dashed vertical line corresponds to the date of guideline release (March 16, 2016). The figure shows the average MME prescribed per day overall and in each surgery category in the initial prescription filled within 7 days after surgery

© 2021 Sutherland TN et al. *JAMA Network Open.*

eReferences

1. Stepan JG, Sacks HA, Lovecchio FC, et al. Opioid Prescriber Education and Guidelines for Ambulatory Upper-Extremity Surgery: Evaluation of an Institutional Protocol. *J Hand Surg Am.* 2019;44(2):129-136.
2. Overton HN, Hanna MN, Bruhn WE, et al. Opioid-Prescribing Guidelines for Common Surgical Procedures: An Expert Panel Consensus. *J Am Coll Surg.* 2018;227(4):411-418.
3. Michigan Opioid Prescribing Engagement Network (OPEN). Prescribing Recommendations. 2020; https://michigan-open.org/prescribing-recommendations/. Accessed June 14, 2020.
4. Hill MV, McMahon ML, Stucke RS, Barth RJ, Jr. Wide Variation and Excessive Dosage of Opioid Prescriptions for Common General Surgical Procedures. *Ann Surg.* 2017;265(4):709-714.

© 2021 Sutherland TN et al. *JAMA Network Open.*
